# Validation of Bakas Caregiving Outcome Scale for brazilian portuguese*

**DOI:** 10.1590/1518-8345.3191.3249

**Published:** 2020-04-17

**Authors:** Tatiana Ferreira da Costa, Cláudia Jeane Lopes Pimenta, Maria Miriam Lima da Nóbrega, Maria das Graças Melo Fernandes, Jordana de Almeida Nogueira, Kátia Neyla de Freitas Macedo Costa

**Affiliations:** 1 Universidade Federal de Pernambuco, Vitória de Santo Antão, PE, Brazil.; 2 Universidade Federal da Paraíba, João Pessoa, PB, Brazil.; 3 Scholarship holder at the Coordenação de Aperfeiçoamento de Pessoal de Nível Superior (CAPES), Brazil.

**Keywords:** Validation Studies, Reproducibility of Results, Nursing Methodology Research, Stroke, Caregivers, Nursing, Estudos de Validação, Reprodutibilidade dos Testes, Pesquisa Metodológica em Enfermagem, Acidente Vascular Cerebral, Cuidadores, Enfermagem, Estudios de Validación, Reproducibilidad de los Resultados, Investigación Metodológica en Enfermería, Accidente Cerebrovascular, Cuidadores, Enfermería

## Abstract

**Objective:**

to analyze the psychometric properties of the adapted version of Bakas Caregiving Outcome Scale for Brazilian Portuguese.

**Method:**

this is a cross-sectional methodological study conducted with 151 informal caregivers of people with cerebral vascular accident sequelae enrolled in Family Health Units. To assess reliability, Cronbach’s alpha was used. Construct validity was verified through exploratory factor analysis, confirmatory factor analysis and correlation with measures of instruments that evaluate correlated constructs.

**Results:**

Cronbach’s alpha for the total BCOS score was 0.89. Factor and exploratory analysis generated a one-factor structure, which was confirmed by confirmatory factor analysis. Construct validity was supported by the high positive correlations with Negative Affect (r = 0.51) and Negative Experience (r = 0.47) of the Well-being Scale and the Depression Anxiety and Stress Scale -21 (r = 0.53) and negative correlations with Positive Affect (r =-0.47) and Positive Experience (r = -0.17) of the Well-being scale.

**Conclusion:**

Bakas Caregiving Outcome Scale shows evidence of satisfactory reliability and validity in family caregivers of cerebral vascular accident survivors.

## Abstract (pt)

**Objetivo:**

analisar as propriedades psicométricas da versão adaptada da *Bakas Caregiving Outcome Scale* para o português do Brasil.

**Método:**

trata-se de um estudo metodológico, de corte transversal, realizado com 151 cuidadores informais de pessoas com sequela de acidente vascular encefálico cadastrados em Unidades de Saúde da Família. Para avaliar a confiabilidade, foi utilizado o alfa de *Cronbach*. A validade de construto foi verificada por meio da análise fatorial exploratória, análise fatorial confirmatória e correlação com medidas de instrumentos que avaliam construtos correlacionados.

**Resultados:**

o alfa de *Cronbach* para a pontuação total da BCOS foi de 0,89. A análise fatorial e exploratória gerou uma estrutura de um fator, que foi confirmada pela análise fatorial confirmatória. A validade de construto foi apoiada pelas altas correlações positivas com Afeto Negativo (r = 0,51) e Experiência Negativa (r = 0,47) da Escala de Bem-estar e com a Escala de Ansiedade, Depressão e Estresse-21 (r = 0,53) e correlações negativas com Afeto Positivo (r = -0,47) e Experiência Positiva (r = -0,17) da escala de Bem-Estar.

**Conclusão:**

a *Bakas Caregiving Outcome Scale* apresenta evidências de confiabilidade e validade satisfatórias em cuidadores familiares de sobreviventes de acidente vascular encefálico.

## Abstract (es)

**Objetivo:**

analizar las propiedades psicométricas de la versión adaptada de la *Bakas Caregiving Outcome Scale* para el portugués de Brasil.

**Método:**

se trata de un estudio metodológico, de corte transversal, realizado con 151 cuidadores informales de personas con secuela de accidente vascular encefálico, registrados en Unidades de Salud de la Familia (SUS). Para evaluar la confiabilidad fue utilizado el alfa de *Cronbach*; la validez de constructo fue verificada por medio del análisis factorial exploratorio, análisis factorial confirmatorio y correlación con medidas de instrumentos que evalúan constructos correlacionados.

**Resultados:**

el alfa de *Cronbach* para la puntuación total de la BCOS fue de 0,89. El análisis factorial exploratorio generó una estructura de un factor, que fue confirmado por el análisis factorial confirmatorio. La validez de constructo fue apoyada por las altas correlaciones positivas con Afecto Negativo (r = 0,51) y Experiencia Negativa (r = 0,47) de la Escala de Bienestar y con la Escala de Ansiedad, Depresión y Estrés-21 (r = 0,53) y correlaciones negativas con Afecto Positivo (r = -0,47) y Experiencia Positiva (r = -0,17) de la escala de Bienestar.

**Conclusión:**

la *Bakas Caregiving Outcome Scale* presenta evidencias de confiabilidad y validez satisfactorias en cuidadores familiares de sobrevivientes de accidente vascular encefálico.

## Introduction

The caregiver is essential to provide the individual with autonomy, independence, inclusion, in the family and social setting, and to avoid hospital readmissions^(^ 1 ^)^. However, if he/she is not prepared to care, it may hinder the engagement of healthy behaviors of the patient and delay patient’s rehabilitation^(^ 2 ^-^ 3 ^)^.

In the case of cerebral vascular accident (CVA) - stroke patients, after being discharged, when affected are commonly dependent on the care of others, which can be performed by a professional called a formal caregiver or by an informal caregiver, who is usually a member^(^ 1 ^)^. This person presents specific and different care needs, such as physical help (moving to the bathroom, to the bed); communication (verbal and nonverbal cues to other family members when the patient has aphasia); support for eating, taking care of their personal hygiene and emotional support (dealing with the destructive behavior caused by the consequences of the disease)^(^ 4 ^)^.

The many tasks that are attributed to the family caregiver, lack of support, unpreparedness to care, the level of dependence of the patient, the chronicity of the disabling situation, the complexity of the care activities, the worsening of health status and the uncertainty of future causes burden, and this may lead to social isolation, reduction or end of leisure activities, impairment of professional activity, loss of job and lack of time for self-care^(^ 5 ^-^ 8 ^)^.

Burden is seen as a multidimensional phenomenon that affects various dimensions of the caregiver’s life, which is defined as the subjective perception that results in the impact of one or more of the physical, psychological, social and financial dimensions resulting from an imbalance between demands that are imposed and the resources available to face them, being a continuous process, usually starting with a certain triggering event^(^ 9 ^)^.

Burden screening is critical for planning psychoeducational and psychotherapeutic interventions to improve the formal support network and caregivers’ ability to cope with the situation in order to improve the quality of life^(^ 10 ^-^ 11 ^)^.

In Brazil, the use of culturally constructed and adapted scales to assess caregiver burden is still incipient, with Caregiver’s Burden Scale^(^ 12 ^)^, Family Burden Interview Scale^(^ 13 ^)^, Zarit Burden Interview^(^ 14 ^)^ and Informal Caregiver Burden Assessment Questionnaire^(^ 15 ^)^. However, there is no specific tool to measure the caregiver burden of people who had a stroke or in other acute and chronic situations adapted to the Brazilian reality.

Two comprehensive reviews of caregiver burden measures^(^ 16 ^-^ 17 ^)^ pointed to the Bakas Caregiving Outcomes Scale (BCOS) as one of the broadest to assess burden. Among its strengths, BCOS takes into account positive aspects of care delivery and its consequences, it is brief, has good consistency, moderate correlations with variable criteria and evidence of good content and construct validity^(^ 16 ^)^.

It was based on the adaptive outcomes of the Lazarus stress and coping model^(^ 18 ^)^. Provision of care and the new roles are considered stressors, however, the caregiver’s assessment of this event is individual and may or may not be perceived as a burden situation. This means that caregivers may experience similar situations, but perceive them in different ways^(^ 19 ^)^.

Given this, the cross-cultural adaptation of BCOS to the Brazilian reality allows the availability of tool that assesses the burden of caregivers of people in situations such as stroke. This scale has high sensitivity to detect changes in self-esteem and financial, emotional and social aspects that have occurred over time. Moreover, it is one of the few developed under the guidance of a conceptual model for developing the items and validation testing^(^ 19 ^)^.

BCOS was made in the United States of America (USA) in English and then adapted and validated for other countries such as Turkey^(^ 20 ^)^ and Greece^(^ 21 ^)^. The use of BCOS has been relevant to practice and research in various areas of health, including nursing, being used, for example, in intervention studies to evaluate the effectiveness of the intervention performed to the caregiver^(^ 22 ^)^. Due to linguistic and cultural differences, it needs to be translated and cross-culturally adapted for use in Brazil by nurses and other professionals.

Considering the above, the following hypothesis was raised: BCOS adapted to the Brazilian context is valid and reliable to assess the burden of caregivers of patients with CVA sequelae. Thus, the aim of this study is to analyze the psychometric properties of the adapted version of BCOS for Brazilian Portuguese.

## Method

This is a methodological and cross-sectional study that analyzed the validity and reliability of BCOS after its semantic and content adaptation for use in Brazil. Authorization for the process of cross-cultural adaptation of the scale was obtained from the tool’s lead author.

The study population consisted of primary informal caregivers of stroke patients. The sample was defined based on Pasquali^(^ 23 ^)^, which suggests that for an appropriate sample for the validation of measuring instruments, at least ten subjects per item should be collected. In this case, as BCOS is composed of 15 items, the sample consisted of 151 subjects.

The inclusion criteria established in this research were: to be 18 years old or older and to be the primary informal caregiver of patients with stroke sequelae registered at USF in João Pessoa-PB. Caregivers who provided care for less than six months were excluded from the study.

Data were collected from September 2017 to December 2017 through individual interviews conducted at caregivers’ homes. At first, a random selection of family health units (USF) was performed, and later, the team and nurses of each USF chosen were contacted, requesting, through the records, the patients who had stroke with sequelae, identifying their caregivers. There were no refusals to participate in the study.

For data collection, the following tools were used: sociodemographic characterization, adapted version of BCOS, Bianchi Stress Scale (EBS) and Depression Anxiety and Stress Scale -21 (DASS-21). The original BCOS is a one-dimensional tool that analyzes the changes in the life of these patients’ caregivers, based on the concepts of social role, subjective well-being and health. It was made with 48 items and subsequently the short version with ten items was defined^(^ 24 ^)^. In the most current version, five items were included, totaling 15 items, measured on a seven-point response scale (“changed for worse” = -3 up to “changed for better” = +3), in which the lower the score, the greater the burden^(^ 19 ^)^.

For convergent validation, the Depression Anxiety and Stress Scale-21 (DASS -21) was used. The DASS was developed in 1995^(^ 25 ^)^ and adapted and validated for Brazil in 2014^(^ 26 ^)^. The tool has 21 items, which are distributed in three four-point, self-responding Likert-type subscales. Each subscale is composed of seven items designed to evaluate the emotional states of depression, anxiety and stress^(^ 26 ^)^.

For discriminant validation, we used the BES scale, which was developed in 1980^(^ 27 ^)^ and validated for Brazilian Portuguese in 2016 (28). It has 23 questions answered on a five-point Likert scale, ranging from total disagreement to full agreement. It is divided into four factors: positive affects - PA; negative affects - NA; positive experiences - PE; negative experiences - NE, and can find a total well-being score calculated by the formula: BESG = (PA-NA) + (PE-NE) as well as specifying negative (NA-NE) and positive (PA) well-being - PE)^(^ 28 ^)^.

For the adaptation of BCOS, the steps recommended by the literature were considered^(^ 23 ^)^, being described as follows: the original version of BCOS was first translated into Brazilian Portuguese by two bilingual translators; subsequently, the translation back to the original language was made and the translated versions were consolidated. This first version was considered by a committee of five judges for the analysis of semantic, cultural, idiomatic and conceptual equivalences, in order to prove the validity of face and content. In addition, simultaneously, the semantic analysis was performed by three people with low and middle education.

Due to the difficulties by the population in understanding the items of the scale verified in the semantic analysis and the low level of agreement among the judges, we chose to use more detailed description of each item based on the suggestions made by the judges between parentheses. This phase was attended by a psychologist, an expert on the subject and the researcher. The psychometric properties of this version adapted for Brazilian Portuguese were analyzed with 151 caregivers of people with stroke sequelae.

Reliability was analyzed using the internal consistency of the scale and the items, using Cronbach’s alpha coefficient, and appropriate results were those that reached from 0.70 to 0.90^(^ 29 ^)^. Construct validity was verified by exploratory factor analysis (EFA), confirmatory factor analysis (CFA) and the correlation between the measures of instruments that evaluate correlated constructs.

Exploratory factor analysis is used when data behavior is unknown and should be performed whenever an instrument with a new sample is applied. Confirmatory factor analysis is performed when the factor structure is known and it is intended to confirm this structure by regression-based structural equation modeling. According to Pasquali^(^ 23 ^)^, to perform construct validation, it is necessary to follow some steps, which he divides into theoretical, empirical and analytical poles.

Prior to EFA, we used the Kaiser-Meyer-Olkin (KMO) measure general sampling suitability and, per item, the Measure of Sampling Adequacy (MAS), whose required score must be greater than or equal to 0.60 for the overall KMO and greater than or equal to 0.50 per item^(^ 30 ^)^. The results for the general KMO were classified as follows: 0.90 is considered wonderful; 0.80 is meritorious; 0.70 is median; 0.60 is modest; 0.50 is miserable and below that is unacceptable ^(^ 31 ^)^. A hypothesis test was also performed using Bartlett’s sphericity test, which verifies that the covariance matrix is ​​an identity matrix, checking if there are no correlations. In this case, the ideal is that the test be significant and the null hypothesis refuted^(^ 32 ^)^.

EFA was achieved by the Principal Axis Factorization (PFA) method. The composite reliability (CR) and the average variance extracted (AVE) were also calculated. In the first indicator, the score level is required to be above 0.70, while in the second indicator a level above 0.50 is required^(^ 30 ^)^.

CFA was performed using AMOS GRAFICS 21.0, considering the maximum likelihood method. The following adjustment indicators were considered^(^ 33 ^-^ 34 ^)^:

- χ^2^ (chi-square) - this indicator checks the probability that the theoretical model will fit the data; in this case, the lower the value, the better. As its use in the literature is low, it is more common to consider the ratio in relation to the degree of freedom (χ^2^ / g.l.). Thus, the maximum value for a proper fit is three;
- Comparative Fit Index (CFI) and Tucker-Lewis Index (TLI) are two indicators that generally compare the estimated and the null model. For this, they consider values ​​close to one as a satisfactory indicator of adjustment, that is: in this case, it is indicated that the scores are higher than 0.90 to say that the intended model represents, in the best way, the construct;
- Goodness-of-Fit Index (GFI) and Adjusted Goodness-of-Fit Index (AGFI) refer to R^2^ in multiple regression, that is, they indicate the proportion of variance-covariance explained by the model from the data. The indicated values ​​are greater than 0.90;
- Root-Mean-Square Error of Approximation (RMSEA) is an index whose values ​​must be less than 0.05 and, in the case of large samples, the value 0.08 is accepted. This index has a confidence interval of 90% (CI90%), which is considered a good indicator for high values, indicating that the model is not well adjusted;
- Convergent validity was performed using the DASS-21 scale and discriminant validation with the BES Scale. These correlations were verified by calculating Pearson’s linear correlation coefficient. The study was developed according to the Brazilian National Health Council Resolution 466/2012, approved by the Research Ethics Committee of the Center for Health Sciences of the Federal University of Paraíba according to the process n. 2,243,225.

## Results

Among the 151 caregivers, the majority (118 = 78.1%) were female, aged between 56 and 65 years (42; 27.8%), married or in a stable union (99; 65 , 6%), with five to eight years of education (41; 27.2%), with an individual income of up to *R*$ 880.00 (67; 44.4%) and family income between *R*$ 881.00 and *R*$ 1760.00 (63; 41.7%) The main source of income was retirement (49; 32.5%) and participants did not consider their income sufficient (88; 58.3%).

Regarding the internal consistency of the items in the Brazilian version of BCOS, a Cronbach’s alpha of 0.89 was obtained for the scale as a whole. The alpha values ​​for the domains ranged from 0.88 to 0.90. Regarding the adequacy of general and item sampling, KMO = 0.872 and MAS were obtained, respectively, with values ​​between 0.794 and 0.919. Also, Bartlett’s test was significant x [χ^2^ (120) = 1135.93; p≤0.001].

Thus, the PFA was performed, which initially extracted three factors with an eigenvalue greater than one, as recommended by the Kaiser-Guttman criterion^(^ 35 ^)^. This three-factor structure explained 57.816% of the total variance. However, it could be verified that the eigenvalues ​​of factors two and three are lower than the values ​​of the parallel analysis (Table 1).

**Table 1:** Eigenvalues, explained variance and parallel analysis for Bakas Caregiving Outcomes Scale. João Pessoa, PB, Brazil, 2017 (n=151)

Factor	Eigenvalue	Variance percentage	Cumulative percentage	Parallel Analysis
1	6.382	42.546	42.546	1.57
2	1.257	8.377	50.923	1.44
3	1.034	6.893	57.817	1.33

From the consideration of these criteria, it was decided by the single factor structure, in which the 15 items factor with loads above 0.40, whose factor explains 42.5% and the commonality. What the items have in common with each other ranged from 0.167 to 0.505. The factor burdens ranged from 0.40 to 0.711 (Table 2).

**Table 2:** Factor burdens and commonality of Bakas Caregiving Outcomes Scale Items. João Pessoa, PB, Brazil, 2017 (n=151)

Items	Burden	Commonality
15. My overall health (State of complete physical, mental and social well-being and not just absence of disease)	0.705	0.498
8. My emotional well-being (Thoughts of joy and pleasure in my experiences)	0.701	0.545
7. My energy level (Readiness to perform daily activities)	0.709	0.503
5. My relationship with friends (Affection relationship)	0.711	0.505
3. My time for family activities	0.677	0.458
4. My ability to deal with stress (Situations that I perceive as threatening)	0.641	0.411
14. My physical performance (My muscle strength, without body aches for daily activities)	0.641	0.411
1. My self-esteem (What I think about myself, my emotions and my behaviors in life)	0.613	0.376
6. My vision of the future (Ability to plan for the near or far future)	0.640	0.410
10. My time for social activities with friends		
9. Social roles (Being a mother or a father, a wife/husband, a sister/brother, a friend, a daughter/son)	0.608	0.370
2. My physical health (General condition of the body in relation to disease and physical ability to perform daily activities)	0.579	0.336
11. My relationship with relatives (Affection relationship)	0.530	0.281
13. My relationship with the patient with stroke sequel (Affection relationship)	0.418	0.174
12. My financial stability (Organization with expenses, control of money, spending, savings)	0.403	0.162
Eigenvalue = 6.382 Variance explained = 42.546%		

The one-dimensional theoretical model of the original version of the scale was tested by the CFA, using data from 151 caregivers. The results showed the following psychometric indicators: [χ^2^ (78) = 91.23; p-value = 0.145; GFI = 0.91; RMSEA (range) = 0.034 (0.03-0.08)]; incremental adjustment measurements [IAM = 0.92; TLI = 0.98; AGFI = 0.90]; parsimonious adjusted goodness of fit (PGFI) [χ^2^/gl = 1.69; PGFI = 0.568] .All saturations (Lambdas, λ) were within the expected range |0 - 1|, which were statistically different from zero (*t* > 1.96, *p* ≤ 0.05). Also, were observed positive Lambda associations (λ) between the factor and its respective items (ranging from 0.42 to 0.75) and WC values ​​= 0.92 and the AVE = 0.66 (Table 3).

**Table 3:** Factor Structure of Bakas Caregiving Outcomes Scale. João Pessoa, PB, Brazil, 2017 (n = 151)

Items	Burden construct			
	λ*	ε^†^	CR^‡^	AVE^§^
1. My self-esteem (What I think about myself, my emotions and my behaviors in life)	0.64	0.41	0,91	0,66
2. My physical health (General condition of the body in relation to illness and physical ability to perform daily activities)	0.54	0.26		
3. My time for family activities	0.66	0.40		
4. My ability to deal with stress (situations that I perceive as threatening)	0.66	0.44		
5. My relationship with friends (Affection relationship, friendship, love, loyalty and protection)	0.71	0.51		
6. My vision of the future (Ability to make plans in the near or distant future)	0.62	0.38		
7. My energy level (Readiness to perform daily activities)	0.70	0.50		
8. My emotional well-being (Thoughts of joy and pleasure in daily experiences)	0.75	0.57		
9. Social roles (Being a mother or a father, a sister/brother, a friend, a daughter/son)	0.65	0.42		
10. My time for social activities with friends	0.59	0.35		
11. My relationship with my family (Affection relationship, friendship, love, loyalty and protection)	0.55	0.31		
12. My financial stability (Organization with expenses, control of money, spending, savings)	0.42	0.18		
13. My relationship with the stroke survivor (Relationship of affection, friendship, love, loyalty and protection)	0.42	0.17		
14. My physical performance (My muscle strength, without body aches for daily activities)	0.55	0.30		
15. My overall health (state of complete physical, mental and social well-being and not just absence of disease)	0.68	0.46		

* λ =Factor Scores of the Structure;

§ ε = Structure Measurement Errors;

‡ CR = Composite Reliability;

§ AVE = Average Variance Extracted. BCOS mean = 48.62; Standard deviation = 12.790. All BCOS items ranged from one to seven and had medians = 4.0 (Possible range 1-7, item midpoints = 4.0, higher scores mean greater positive changes)

**Figure 1:**
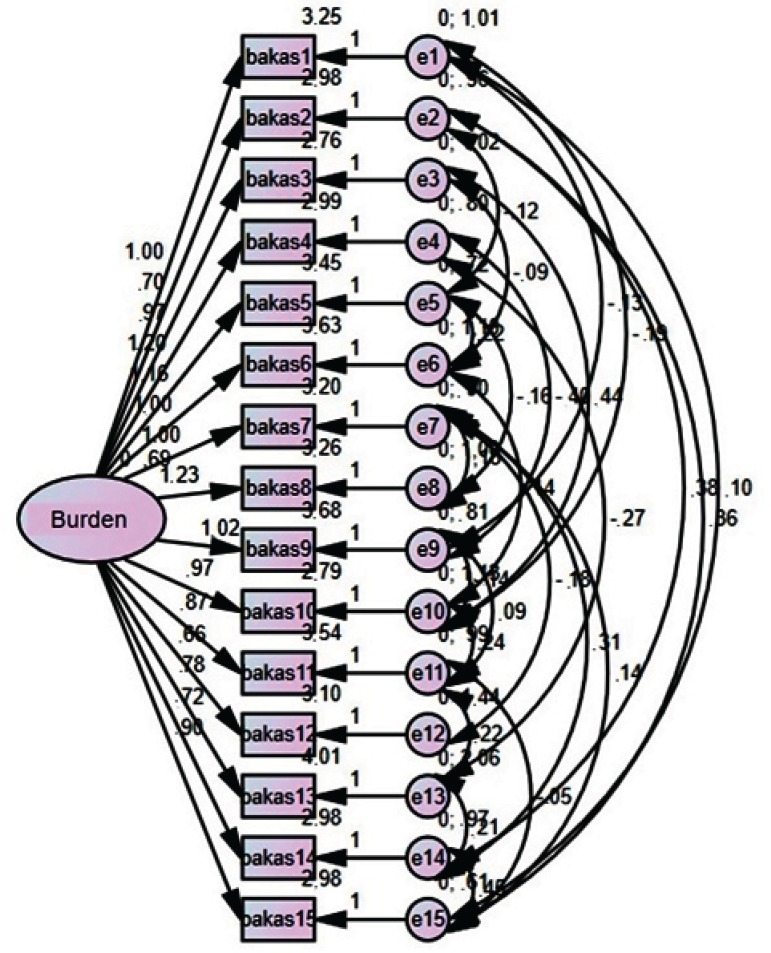
Path diagram for burden. João Pessoa, PB, Brazil, 2017 (n = 151)

Source: AMOS version 21.0

The factor structure of the BCOS scale for burden was adequate and robust for the evaluation of this construct (Figure 1).

In the divergent analysis, BCOS was negatively correlated with BES Positive Affect (r = -0.47) and BES Positive Experience (r = -0.17). And, in the convergent analysis, there was a positive correlation with BES Negative Affect (r = 0.51) and BES Negative Experience (r = 0.47) and with DASS-21 (r = 0.53). All results were significant p ≤ 0.05 (Table 4).

**Table 4:** Convergent and divergent analyzes of Bakas Caregiving Outcomes Scale. João Pessoa, PB, Brazil, 2017 (n = 151)

Factors	BCOS*
Subjective well-being	
Positive	
Positive Affect	-0.50
Positive Experience	-0.17
Positive Well-being	-0.41
Negative	
Negative Affect	0.51
Negative Experience	0.47
General Well-being	-0.47
Stress, Anxiety and Depression (DASS-21)	0.56

* BCOS = Bakas Caregiving Outcomes Scale

## Discussion

Burden is a multidetermined phenomenon that occurs when care demands are greater than available resources. BCOS evaluates the burden through changes in the caregiver’s life after an acute or chronic event, such as stroke, and, in its original version was reliable and valid to evaluate the construct^(^ 19 ^)^.

The adaptation and validation process of BCOS -15 items went through the steps suggested in the literature^(^ 23 ^)^ that involved translation, translation synthesis, back translation and semantic and content validation with the target population and judges, respectively, and pretest.

The cultural suitability of a translated tool enables its applicability and functionality to be equivalent to the original instrument in its respective country by clarifying the obscure points present in the translated text. This aspect improves interaction and communication during the search for information to be evaluated^(^ 36 ^)^.

Regarding the reliability of the adapted scale, the internal consistency performed by Cronbach’s alpha was 0.89, which reveals an internally consistent measurement. This value was similar to the alpha of 0.90 from the original version^(^ 19 ^)^ and also, in another BCOS validation study in caregivers of patients with cancer, alpha was 0.83^(^ 21 ^)^. In addition to Cronbach’s alpha, these two studies performed the test-retest reliability analysis, which revealed good stability after two weeks.

In order to explore the dimensionality of the scale, the EFA was performed, aiming to extract the maximum number of factors^(^ 37 ^)^. Initially, three factors were extracted, however, the items factored in more than one factor after rotation, which conceptually, makes no sense. In addition, two factors had an eigenvalue score below the recommended. Thus, it was decided to maintain the proposed unifactorial structure on the original scale^(^ 24 ^)^.

The variance explained by the single factor was 42.54%; the commonality of the items from 0.167 to 0.505 and the factor burdens from 0.40 to 0.711. Similar results were presented in the original BCOS validation with 147 caregivers, where there was 42.8% of the variance represented by the first factor and the factor burdens ranged from 0.41 to 0.78^(^ 19 ^)^.

Regarding factor burdens, most items factored above 0.5, a value recommended by the literature^(^ 30 ^)^, except for the items Relationship with the patient (0.418) and Financial stability (0.403). The latter was included in the 12-item BCOS through two alternatives: “My ability to buy basic necessities items” and “My ability to pay bills”, which were excluded for having unsatisfactory burden loads. Subsequently, the item was reformulated and included in BCOS 15 items as “Financial stability”. In this study, we chose to leave on the scale.

The financial impact on caregivers’ lives is a common stressor due to expenses with care, diaper, medical appointments, medication, rehabilitation therapy, and private transportation to take patients to health facilities and hospitals. Moreover, they often have to quit their jobs and, consequently, lose their income and become totally dependent on the financial support of other family members, who may stop it regurlaly^(^ 38 ^)^.

The one-dimensional theoretical model of the original version was tested by the EFA. The results of this analysis showed a good fit of the BCOS adapted version for Brazil with strong correlations between scale items. This version can be considered adequate and valid, considering these indicators, for what it is intended to measure in the referred sample. AVE and CR also presented satisfactory results, which evidence both the reliability and the convergent validity of the evaluated construct, which justifies the adequacy of the factor structure of the aimed measure reliably and with factor security.

In the most current scale validation studies assessing caregiver burden, such as the Caregiver Burden Inventory^(^ 39 ^)^ and the Informal Caregiver Burden Assessment Questionnaire^(^ 15 ^)^, similar tests were used, such as Cronbach’s Alpha and correlation with other constructs and CFA. However, in both cases, EFC was not used to explore how many existing factors and also the number of indicators in the CFA was lower than this study. In the referred studies, the number of dimensions were different from BCOS, composed respectively by five and seven factors.

Regarding convergent and discriminant validation, BCOS was positively correlated with DASS-21 and BES negative affect and negative experience dimensions and negative correlation with BES positive affect and positive experience dimensions. Studies have shown that physical, emotional, financial and social burden resulting from the caregiver role has been associated with mental disorders such as depression, anxiety and stress^(^ 40 ^-^ 42 ^)^, which consequently affects well-being and quality of life^(^ 43 ^)^. A systematic meta-analysis review found that caregivers of stroke patients showed a doubled risk of psychic symptoms compared to the general population, with overall prevalence of 40.2% and 21.4% of depression and anxiety symptoms, respectively^(^ 44 ^)^.

For this reason, it is important to perform family-specific nursing interventions during hospital discharge, immediate post-discharge and over time, with psycho-educational therapies, skills training and therapeutic counseling, which will help to reduce anxiety and burden and to have a more favorable outcome. Studies have shown that caregivers’ needs are not stable through the different phases after stroke^(^ 45 ^)^.

The limitations of this study were: restriction of research with only caregivers of individuals with stroke, making it impossible to evaluate its effectiveness in other caregivers, such as children who have a disease, people with mental disorders, cancer, among others. In addition, the generalization of the results is limited to caregivers residing in only one geographic region of Brazil, who have specific habits and culture, which may influence the tool’s responses.

## Conclusion

The 15-item BCOS provides satisfactory evidence of reliability and validity in family caregivers of stroke survivors, and one-dimensionality was supported by CFA pointing to a good fit index. BCOS validation for Brazilian Portuguese is promising for research with this population, as it is sensitive enough to detect changes in life and differences between groups in intervention studies. In addition, as a short and easy-to-manage tool, it can be a valuable assessment tool for nurses to identify deteriorating aspects of caregiver’s life as a result of care, and to identify priority areas for interventions as well as to assess and document their progress over time.
